# A Report upon the Latest Achievements in the Sphere of Epilepsy, from the German of Dr. Finkelaburg, of the University of Bonn, Germany

**Published:** 1863-12

**Authors:** 


BUFFALO
### Medical and Surgical Journal.

VOL. III. DECEMBER, 1863. NO. 5.

ART. I.—*A Report upon the latest Achievements in the Sphere of Epilepsy, from the German of Dr. Finkelaburg, of the University of Bonn, Germany. Translated, and with notes by* H. Lassing, M. D., *Physician and Surgeon to the Eastern Dispensary, New York.*

For the Buffalo Medical and Surgical Journal.

The latest progress of science in epilepsy and convulsive affections in general, have their most important starting point in the developments of the *theory of reflex movements;* and we must acknowledge Marshall Hall to be the real founder and most assiduous laborer in the field, a merit, the importance of which cannot be in the least obliterated by the error into which this inquirer was led by his attempts to explain these phenominæ.

But Hall was not satisfied with his physiological observations of these reflex actions, but the intimate relation of these to *Epilepsy* was so far from escaping his attention, that as early as 1851. in the *Academie des Sciences* he dared to make the proud assertion, that through his researches he had reached near to that point, which Esquirol placed beyond the possible reach of human undertaking, namely, a physiological mastery of epilepsy. If this and some other expressions Hall made, looks like a species of over-estimation of the practical bearings of his services, we must nevertheless credit the physiologist with them. Neither are his erroneous explanations of some of the symptoms which constitute the epileptic attack, and the consequent clinical deductions, the errors of which we will now illustrate, to make the pathologist forget that the insight into *reflex neuroses* now become so important, and science and practice remains indissolubly connected with the remembrance of Marshall Hall as its creator.

In 1855 Hall published a comprehensive treatise, consisting of his observations and the theories based thereon upon the part which reflex action plays in pathology, which he had previously singly communicated to the Academy, (Hall, Marsh, Aperçu du systéme spinal on de la série des actions rèflexes dans leurs applications á la Phyiologie, á la Pathologie, et spèlialement á l'Epilepsie. Paris: 1855, Masson.) In this work he developed the following upon Epilepsy:

1st.—The disease consists either of a disease of texture of the spinal nerve-centre, ("épilepsie organique,") or of a morbid increase of the reflex excitability of this nerve-certre, arising and kept up from without ("épilepsie inorganique.") (It is only to the latter and more frequent form which stands parallel to simple paroxismal Apoplexy and intermittent mania that Hall's researches have relation.)

2d.—The causes of epilepsy, without disease of texture of the spinal marrow (the épilepsie inorganique) are either emotions arising from the brain, which propagate themselves upon the spinal marrow—powerful mental affections or visceral, gastric, sexual, or finally irritations originating in the peripheries which put the spinal marıow into a morbid state of excitement. In all these cases the epileptic convulsions are reflex, or according to Hall's nomenclature "diastaltic."

3d.—The motor reflex action is always first exhibited in the external cervical muscles, such as trachelismus, sometimes in the shape of torticollis, or with participation of the inner laryngeal muscles, as laryngismus. After this only will the compression of the cervical veins and larynx, produced by the convulsions of the cervical muscles, affect the brain and result in suspension of sensation and general convulsions.

4th.—The last named and most dangerous symptoms of epilepsy can be done away with, or at least in all cases materially mitigated by tracheotomy. Against the accusation that he recommended this as a remedial agent in the disease, Hall defends himself with determination, and says he never expected any but palliative results from it; nevertheless he certainly believed that through this method many a patient was not only protected against danger of losing life, but was also saved from the reaction of severe epileptic paroxysms upon the brain—epileptic idiocy.

5th.—As to the radical treatment of the disease itself, Hall recommends in the first place a strict avoidance of all causes tending to excite reflex action, physically as well as mentally, to do which a careful reform of the entire mode of life is necessary; and secondly, a reduction of this excitability of reflex action itself by well regulated corporeal exercise and the careful use of gradually increased doses of the Extract of Hyoscyamus.

The practical, true conclusions of Hall's theories of epilepsy, culminated in the new views of trachelismus, and particularly laryngismus, the correctness of which he sought to defend against many heavy assaults. Then he looked for other conditions in which these reflex cervical convulsions came under observation. He found the same in *tetanus*, *hydrophobia* and *poisoning by strychnine*. While studying the physiological relations of the latter experimentally, he believed to have observed that strychnine produced death by laryngismus, and in fact that tracheotomy would entirely prevent death, or at least materially prolong life. As a further practical corroboration of his teachings, Hall in the appendix of his book, gives eight cases where severe epilepsy was reputed to have been successfully treated by tracheotomy, by different English and American physicians. What value we are to place upon them the following analysis will show:

In the first case (reported by Dr. Cane. Uxbridge,) the patient was free from attack for seven months after the operation—when patient became lost to view. Regarding the previous frequency of the paroxysms there is no account.

In the second case (by Dr. Macharsie. Chesterfield,) the operation resulted in considerably mitigating the attack; after cicatrization of the tracheal opening, they returned with double violence, and patient died during a paroxysm.

The third patient (by Dr. Niel. Philadelphia,) had at first after the operation only incomplete, but after three months' much severer attacks of the disease, and succumbed to one without any closing of the tracheal opening or any other influences being exercised upon the latter.

A fourth observation (by Dr. Herrick. Chicago,) referred to a case who died on the second day after operation, but Hall believes that it would have resulted favorably had the operation been made earlier.

The fifth patient (operated upon by Dr. Bucknill,) was at the Devon County Asylum. The attacks returned half as often and two-thirds as violently as before the operation, but patient soon died from a pulmonary affection.

In a sixth case, (a lady, operated upon by the same physician,) there was an unmistakable amelioration of the convulsive attacks, as well as an improvement of the mental health; but after nine months—with an undisturbed tracheal opening—had a much more aggravated relapse, ending in death.

In the seventh case (observed by Dr. Edwards. Cheltenham,) there was considerable amelioration of the paroxysm, during the six weeks the canula remained in the wound; after its removal, violent attack with fatal coma.

The eighth and last observation related to an eclamptic case, is one in which tracheotomy procured a momentary improvement of symptoms of suffocation and improvement of the general convulsions, yet could not prevent a fatal end in a very few hours.

These are the eight out of the twelve operations in tracheotomy for epilepsy had up to 1855, which Hall "selects for communication." That the four cases which were not "communicated" did not result more favorably can be taken as a foregone conclusion, from the fact that Hall silently passed over them. And upon such, more than doubtful results, he demands a repetition of an operation, in itself dangerous to life as a palliative in a disease which in itself is seldom fatal. Notwithstanding this, Hall's authority, after the appearance of these reports, induced further experiments, two of which were fully described by two English physicians, J. W. Ogle and Edward Jackson Riccard.

The first performed tracheotomy on a boy aged sixteen years, who had been five years afflicted with epilepsy, and latterly had several attacks daily, and already showed unmistakable signs of commencing idiocy. Before and after the operation the administration of various vermifuges resulted in the discharge of a great mass of ascari oxyuræ. The attacks (the tracheal opening being kept so) gradually were reduced to one attack per day which was not at all accompanied with the former troubles of suffocation. Besides the constant wearing of the canula, vermifuges and general tonic were administered for several months, and the boy who also showed more mental freedom and awakening, was ordered to take a trip to Brighton. A year afterward his condition became worse, the paroxysms were more frequent and violent, and the boy often remained insensible even during the intervals of paroxysm. The canula which was removed during a paroxysm could not again be returned. and with the healing up of the opening there was not only no relapse, but on the contrary observable improvement of his general condition and amelioration of the spasms resulted.

The physician to the Brighton Hospital, Dr. Ormerod, who last observed the patient, attributes the temporary favorable effects of tracheotomy, principally to the psychical impression and is of opinion, that any other severe operation would have had a similar success.

As a proof of the inefficiency of tracheotomy Ormerod quotes a case of epilepsey, in which an abscess of the glottis necessitated the aforesaid operation, and which nevertheless succumbed the following day to a severe attack.

Dr. Ricard (*Lancet*, June, '59,) opened the trachea in a female aged 37 years, an epileptic. During six months when the canula was worn, the attacks were milder, but on the other hand, from the day of operation the patient suffered from hæmoptjis. In seven months,after previously laying aside the canula, the former violence of the affection returned and an unusually vehement paroxysm, (though the canula had been replaced,) ended fatally.

Notwithstanding the slight encouragement which such results could give, the method was nevertheless not left without trial in Germany.

Dr. C. Westphall, of Berlin, reports (Annals Berlin Charitè,) an operation of tracheotomy performed by request of Jader upon a patient, whose condition, according to the accompanying description, deserves more to be called hysteric than epileptic. An apparently danger threatening spasm of the glottis formed the indication for the operation, after which and as soon as the febrile re-action had ceased, the convulsive attacks showed themseives at first milder, then as before with general convulsions and loss of sensibility, notwithstanding unimpaired respiration through the canula.

That a spasmodic closing of the glottis increases the importance and danger of the epileptic attack and favors a general distribution of the convulsive shock other experience does not permit us to doubt, and therefore the temporary amelioration of epileptic attacks after tracheotomy cannot, as some (Hasse, for instance,) have believed, be ascribed *solely* to the general impression of the operation. A real gain nevertheless, can hardly be expected as the creation of an opening is in itself dangerous and its being kept open difficult, besides to a very great extent permanently exposing the organs of respiration and the accidental injuries to these which can never be surely prevented, may be followed by the worst consequences. Two of the aforementioned patients died in consequence of the pulmonary irritation, while on the other hand the gain to be derived from the operation as this experience proves, is extremely uncertain and limited. As to Hall's argument concerning the independence of the mental symptoms, particularly the insensibility from an asphyxiating return of venous blood to the brain, it is counterbalanced as Foville has already shown, among other things, by the simple fact that the epileptic, at the beginning of the attack, (at the moment of his insensible downfall,) never looks congestive, but on the contrary pale, and the venous congestion and facial bloating only appear during the general convulsions.

Sieveking, whose book (on epilepsy and epileptiform seizures, London, 1858,) is remarkable for a series of excellent observations, believes the cause of our deficient knowledge of epilepsy to be principally our observation, of the most remarkable symptoms, namely: The convulsive attack itself has led us to neglect the permanent symptoms of the disease which continue during the so-called intervals. He claims that epilepsy is a disease of the entire body, not that of a single organ, and that a careful observation of the entire system, and the entire organic functions, but particularly those of the nervous system, are necessary to form a correct judgment, not only for every case of pure epilepsy, but also the relation of collateral circumstances to the epileptic bases. The epileptic attack is the blossom of an injurious weed. Not only the blossom, but also the root and trunk must we learn to know and extirpate. The oftener the paroxysms, the more distinct will we find the symptoms of the intervals, which exhibit themselves in three classes; as an heightened cerebro-spinal reflex irritability, as a diminution of intelligence, and finally as a diminished energy of the sympathetic nervous system.

The increased sensatorial and motor excitability evinces itself by the unsteady aud restive look and expression, a tendency to hurried, trembling movements of a convulsive character, by spasmodic sensations similar to those felt in hysteria, and in excitability of temper, force of the voluntary impulses, in one word, in a general nervousness which often can be plainly read by the first view of the external appearance of the patient. Next we notice a disturbance of the power of mind in an unaccustomed difficulty of associating and combining thoughts; then memory becomes weak and particularly after every attack defective; the first indication of idocy which will snpervene after a while. The sympathetic affection of the brain is also shown by headaches, dizziness, dilation or uneveness of the pupils, and a rapid and weak pulse. Finally, the 3d group of symptoms are held as not unimportant by Sieveking, which indicate a torpor of those organs depending upon the sympathetic nerve; as want of digestion with inclination to dyspepsia, flatulence and constipation, irregularity of the catameniæ. All these symptoms appertaining to the intervals appear in different stages, combined generally, more seldom singly; but entire want of their appearance among fifty cases which Sieveking observed, did not occur. Our knowledge of the predisposing causes of epilepesy is yet very limited and requires a much larger field of statistical observations than those yet had, for which we are solely indebted to negative instruction.

Sieveking also gives a table of comparisons regarding the frequency of the disease in the French and British armies, but this proves no influence of race, nor difference between inhabitants of high and low countries, as has been asserted, yet they do prove a relative endemic frequency in some neighborhoods, without any, as yet, authentic cause. For example, among the French conscripts, aged 21 years, the proportion of epileptics, according to the different departments, varied from 41,5 to 339,0 in 100,000. The lowest proportionate number came from the department Puy de Döme, the highest from the department Pyrénées Orientales. The department De la Seine ranged about half-way, with 137. Such marked differences which are corroborated by official figures as to other localities, as in the dukedom of Nassau (a v. Franque Mass. Med. Jahrbuch,) require a more satisfactory explanation than that given in the suggestion of Sieveking, "that in those departments where the greater number of epileptics came from hereditary influences in the course of time had an overpowering effect." Several cases cited as epidemic epilepsy, such as the accompanying symptoms of dancing mania, and as in 1842, in Teheran, clearly do not appertain to this class, but are to be accredited to the co-ordinate spasms, without any interval of sensibility, the epidemic powers of spreading of which is universally known.

Among the cases of epilepsy observed by Sieveking there were 11⅞ of a direct hereditary nature. With the others, the predisposing causes, as far as they could be ascertained, consisted in such influences which a general debility of the circulation and nervous system usually draws after them; sexual excesses, particularly masturbation, chlorosis. leucorrhœa, physical deprivations, bodily and mental over-exertions, etc.

Upon this ætiological basis of the overwhelming majority of epileptic cases, Sieveking very properly places the most practical value, because in them he sees the safest land-mark for a correct therapeutic method. Exciting causes, susceptible of proof, were observed by Sieveking in one-third of his cases, and consisted mostly of psychical affections, such as fright, terror, great sorrow; next came injuries of the head, otitis, scarlatina, helminthiasis, irritation of dentition, etc. Psychical causes were more frequently observed in female patients (54 per cent.) than in males, (35 per cent.,) a fact which coincides with the general opinion of the greater susceptibility to psychical impressions in the female sex. The true importance of the balance of the so called exciting moments to the cure of epilepsy, is not placed in any new light whatever, by the observations of this author. All the physical disturbances enumerated, happen so extremely often without being succeeded by epileptic symptoms, that we want every standard by which to judge their real significance. The same may be said, according to Sieveking's view, of the pathological state of the nervous centres, in which the causes of epilepsy were thought to be discovered. They all occur a thousand times without any epilepsy, and, regarding the manner of their possible relation to epilepsy, we are, as yet, entirely in the dark. A grand summing up of his experiences, lead Sieveking to adopt a theory of a change in the nervous system of the epileptic patient, or the subject predisposed thereto, the nature of which is, as yet, unknown, which may lay dormant for years, even for life until awakened and brought into action by an adequate irritation, just as different substances have a different degree of inflamability from a jet of flame approaching then, so men vary in their susceptibility to external disturbing influences upon the nervous action. Some are perfectly fire-proof others yield gradually, and still others are as inflamable as tinder. This is also the case, as in most neuroses, in epilepsy. The disease is everywhere the same, and all classifications, according to the different causes, have only a limited value. Even the distinction between idiopathic and symptomatic epilepsy, is considered as so difficult of being carried out, and of so little practical value, that he only sees in it a danger of losing sight of the unity and identity of the form of disease.

Sieveking believes that, the first attack, in many cases, is produced by a disturbance of the intercranial circulation, be it through a diminished or increased supply of blood, (of course, with a pre-existing disposition,) and refers, in addition to his own experience, to the well known experiments of Sir Astley Cooper, who, by litigation of the carotid, and compression of the vertebral arteries produced epileptiform attacks in animals. In some cases bodily and mental over-exertions preceded the first as well as the succeeding attacks,—an observation which would recommend the observance of physical and psychical quiet in the treatment of this disease.

The brain, according to Sieveking, must under every circumstance, be considered as the nearest participating organ in epilepsy, as the suspension of the activity of the brain and of sensibility is the most constant of all symptoms and cannot in any way be explained as an affection of the spinal marrow alone.

In the treatment of epilepsy, S. keeps two objects principally in view: general strengthening of the nervous system, and derivation from the head. The first indication is to be met by bodily and mental quiet, scrupulous regulation of the diet, and particularly by the use of cold baths; the second, by the employment of a seton, purgatives, zinc and other metalic remedies. He advises against the undertaking to treat epilepsy without the use of the cold bath. In treating debilitated individuals, he at first increases the temperature of the bath a little and then gradually lowers it. To augment the re-action in torpid subjects he at first adds salt to the bath. With strong patients he soon gives the cold douche, the depressing influence of which upon the nerves will soon show itself in a considerable reduction of the frequency of the pulse.

As an opposite to Sieveking's book, which contained the observations of the most practical points, the third edition of Radcliffe's Treatise upon epilepsy and other convulsive diseases (Radcliffe, Chas. Bland; Epilepsy and other Convulsive Affections, their Pathology and Treatment, 2d edition; London, 1861: J. Churchill.) created considerable surprise by the originality of the scientific theories it puts forth. Ever since 1855 Radcliffe defended the theory that muscular contraction is not a result of an irritation of the muscular tissue emanating from nerves, but on the contrary represents the normal queit or equalized condition of the same while relaxation is caused and maintained through the influence of the living nerve. The physiological function of a muscle, its contraction, would, according to this, be a species of negative act, as the same could only be voluntarily produced by means of a withdrawal of the relaxing enervations. This paradoxical view, to which however, some time since, Mattendi in France, and Eagel and particularly Stannius in Germany, have become converts, takes its arguments principally from the comparison of an active muscle with those in a condition of dead rigidity, which latter Radcliffe, as well as Stannius, calls the natural condition of every muscle, freed from nervous influence. In both, the electrical muscular current ceases, in both, the elasticity of the muscular substance appears in greater force, while in the relaxed condition they are held by the nervous influence. As further evidences Radcliffe in a full description of his numerous experiments enumerates, the supervening of convulsive movements by cutting off the supply of blood, the disappearance of *rigor mortis*, by the re-establisment of irritability after transfusion of blood into the arteries of the muscles, and finally the fact that the direct incitability of muscles after severing the appertaining nerves regularly increases

The relaxed muscle is thought by Radcliffe to be in a state of polar electrical expansion, which is to be relaxed by specific influences, particularly by galvanic currents, whereupon the muscle as long as the relaxing, freeing influences continue, will relapse into its normal state of contraction. For a time this revolutionary theory did not want supporters, particularly among neuropathologists, who, as well as Radcliffe, saw in this a plausible explanation of many heretofore mysterious circumstances in the field of theories of neuroses, and for this reason were less exacting of physiological proofs.—Under a sober test, however, this shaky fabric could not stand long. It was soon found that in fact, although in death's rigor, the elasticity of muscles is increased, but in activity, is on the contrary very much diminished. (Funke.) It was remembered that the fluctuations, *i. e.* interruptions in the muscular stream were simply results of physical changes, in the muscular substance and does not in any way necessarily prove a ceasing nervous influence "the natural condition of every muscle freed from nervous influence, and finally the observation of Bernard and Kolliker, that after destruction of the extreme nerve-end in a muscle by woorara no rigor supervenes, but that this is the case where the muscle itself is destroyed by veratrine, must be held valid as the most decided disproval of Radcliffe's and Stanius' position.

Nevertheless let us follow Radcliffe in the application of his theory to the elucidation of the convulsive neuroses, particularly epilepsy. First, he believes himself to be able from immediate observation to prove the fact, that the commencement of epileptic convulsions is equally regularly combined with a stoppage of the supply of arterial blood to the head as it was the case in the experiments of Sir Astley Cooper, (and in Germany, Kussmaul and Tenner's) after ligation of the arteriæ vertebræ and compression of the carotids. The deathly paleness of the countenance immediately before and at the beginning of the epileptic paroxysm he considers as conclusive against the adoption of the idea of arterial as well as venous congestion, but is in every way consistent with the laws laid down by this writer in connection with his foregoing theory, that the stimulus of the arterial blood upon the nervous system is not accelerating but obstructing in its relations to the muscular action. If we consider the attack as caused by an obstruction rather than an acceleration of the relations between the arterial blood and brain, we will find a similar state in the interval. He alleges that with most epileptic patients the pulse is weak, slower (?), the temperature of the general surface lower than normal, respiration imperfect and irregular. The entire appearance of the epileptic would indicate rather a reduced than an heightened activity,—the brain particularly psychically indicates the direct opposite of excessive development of power, and this not only during, but in the intervals of the attack. A further corroboration of his views is found by Radcliffe in the pathological appearance of epilepsy, inasmuch as he refers to their corresponding with those of idiots, discolorations, atrophy of the grey substance, chronic softening or induration, hydrops of the ventricles, etc. The reports of Schroeder van der Kolk, (of which we shall speak hereafter,) published in the meantime, prove according to Radcliffe, a diminished activity of the medulla oblongata, and consequently the opposite of that explanation which the discoverer himself would ascribe to them. Again, the expansion of the vessels is not to be referred to arterial hyperæmia, but to a backward movement of venous blood during the attack, and the main cause of the secondary atrophy and degeneration of the substance is to be found in a diminished power of resistance in the latter (?) Original debility or incidental stages of exhaustion of the medullary substance accordingly would represent the pathological foundation of epilepsy, which like other symptoms of so-called "morbid excitability," do not indicate an excess, but a deficiency of enervation. Also other classes of reflex neuroses, such as those of teething, irritation of worms, uterine patients, &c., are viewed by the author as simple states of exhaustion of the nervous centres caused by pain, want of sleep, loss of blood or fluids, and are more or less clearly and nearly regularly exhibited in the functions of the circulation, formation of the blood, and in respiration. And for this very reason (?) the author considers that this entire class of disease without the adoption of his asserted physiological theory of an obstructing muscular enervation is incomprehensible.

The advantageous side of Radcliffe's teachings is probably, that they do not exert any misleading influence upon therapeutics but on the contrary seem to serve as a staunchion to the rules sanctioned by general experience.

Insufficient activity of the nervous system and debility of circulation, viewed as the foundations of the disease, must exclude all influence, tending to weaken the system, and on the contrary indicate a careful regulation of the diet and manner of living generally as well as the exhibition of *tonics*, mild stimulants and such other remedies as we know tend to assist in the building up of the nervous system. These principles are sure of general approval, but less so necessarily, must be the indiscriminating method upon which R. bases his views of the test of remedies recommended in epilepsy. Because fat is a large ingredient in the brain and nerve substance, he advises the food for epileptic patient to be prepared more rich in oils than for those in health, and particularly recommends the use of the brain of animals as a suitable article of diet. Iron, zinc cod-liver oil, oil of turpentine, valerian, camphor, naptha, all were found by him very effectual "in certain cases," the last named remedies, particularly where there was general prostration of strength. Epileptic patients from malarial districts "usefully took quinine." With such having a predominating erotic diathesis, "the bromide of potassium did good service." But the best results "he promises himself" from his latest experience with the combination of phosphorus and cod-liver oil, to the use of which he was led by finding that fat and phosphorus are the very ingredients of the brain, which increase or decrease in exact proportion to its powers. In an accurate analysis of the brain of six children, youths, men, old men and idiots, L'Heritier found the following averages:

| | Children. | Youths. | Men. | Old Men. | Idiots. |
|---|---|---|---|---|---|
| Fat | 3.45 | 5.30 | 6.10 | 4.32 | 5.00 |
| Phosphorus | 0.80 | 1.65 | 1.80 | 1.09 | 0.85 |
| Albumen | 7.00 | 10.20 | 9.40 | 0.65 | 8.40 |
| Osmazone and Salts | 5.96 | 8.59 | 10.19 | 12.18 | 14.82 |
| Water | 82.79 | 74.26 | 72.51 | 73.65 | 70.95 |

According to this it would seem that idiots possess only one-half the average proportion of phosphorus found in the brain of adults, and considering the intimate relationship of idiocy and the more unfavorable forms of epilepsy, a similar relative proportion is to be expected in the latter.—The form in which Radcliffe exhibits phosphorus is the following:

℞ Ol. Phosph. (Pharm Boruss,) ʒi ij.

Ol. Morrh, ℥ vi misce.

S ½ to 1 Table Spoon full 3 times a day.

Radcliffe has also great faith in the curative powers of the constant electric current, as produced, of effective strength, by Pulvermacher's chains, when used in epilepsy, for the especial reason that Matteucei in passing it through the spinal substance of animals poisoned by strychnine, observed no convulsions. Particularly useful, he says, is the employment of the current as a direct preventive of the expected attack as announced by premonitions, or as to be expected from its observed periodicity. This want of even the smallest appearance of science throws a dark shadow upon the general value of Radcliffe's researches, and is the less excusable, inasmuch as the author, according to his own assurance, is a much employed practitioner in the treatment of patients afflicted with epilepsy and nervous diseases generally, and therefore should know from experience, that in this respect, a cautiousness, even distrustful, is far to be preferred to reckless credulity.

Now to turn from this surprising picture of British natural philosophy to the more refreshing labors upon the continent, we find first of all in Germany, regarding the physiological view of epilepsy, and particularly regarding the theory of the immediate seat of the disease, that a firmer basis has been gained through the researches of Pflüger upon the functions of the spinal marrow. Pflüger first proved the fact, that the direction in which sensational excitements are converted into reflex movements, always radiate toward the medulla oblongata, such as from the cerebral nerves downward, from the spinal nerves upward. Only then when the reflex excitement reaches the medulla oblongata can the reflex movements be communicated to the other side and spread over the entire body, while in the brain itself, as well as in the medulla spinalis only one sided reflex movements are caused. This not only proves the medulla oblongata to be more susceptible to sensational excitements, but also that in the medulla we are necessarily to look for the origin of all bilateral, clonic and tonic forms of convulsions. A further axiom of importance to neuropathology in the reflex theories of Pflüger, is that in difference of intensity of convulsions laterally, the intensest one takes place on the side upon which the irritation was applied to the medulla spinalis, or made its exit from the brain.

A brilliant corroboration and filling up to Pflüger's results were found in the observations of Brown Sequard, who succeeded, by injuring the lower part of the spinal marrow, in producing a slow progress of the state of traumatic irritation, to the medulla oblongata, the attack of which (after about three weeks) was shown by bilateral, epeliptiform and periodically remittent convulsions. The latter, however, could be voluntarily produced by irritating single cutaneous branches of the trigeminus on the injured side.

[To be Concluded in next Number.]

